# In-hospital outcomes of ruptured abdominal aortic aneurysms: A single center experience

**DOI:** 10.34172/jcvtr.2022.02

**Published:** 2022-03-06

**Authors:** Niki Tadayon, Mohammad Mozafar, Sina Zarrintan

**Affiliations:** ^1^Division of Vascular & Endovascular Surgery, Department of Surgery, Shohada-Tajrish Medical Center, Shahid Beheshti University of Medical Sciences, Tehran, Iran; ^2^Health policy Research Center, Institute of Health, Shiraz University of Medical Sciences, Shiraz, Iran; ^3^Cardiovascular Research Center, Tabriz University of Medical Sciences, Tabriz, Iran

**Keywords:** Abdominal Aorta, Rupture, Aneurysm, Mortality

## Abstract

***Introduction:*** Ruptured abdominal aortic aneurysm (RAAA) is a catastrophic condition with in-hospital mortalities up to 89%. Patient survival depends on multiple factors; however, prompt surgery is essential to prevent mortality. We report the in-hospital outcomes of RAAA at a high-volume and teaching vascular surgery center in Iran.

***Methods:*** This study is a single-center retrospective analysis of patients with infrarenal RAAA during February 20, 2012 to December 21, 2019 at Shohada-Tajrish Medical Center, Tehran,Iran. We identified 66 patients with RAAA during the study period. The patients were dividedinto two groups based on their transfer status (Transfer group versus non-transfer group). The primary outcome was in-hospital death. The secondary outcomes were in-hospital myocardial infarction (MI), abdominal compartment syndrome (ACS) and postoperative renal dysfunction requiring dialysis.

***Results:*** The mean age of the patients was 74.2 ± 8.3 years. Forty-seven patients (71.2%) were transferred to our center from other institutions. There were 46 in-hospital deaths (69.7%) and three in-hospital MIs (4.5%). Three patients (4.5%) had postoperative ACS and six patients (9.1%)had postoperative renal dysfunction requiring dialysis. Transfer patients had an increased rate of in-hospital death compared to non-transferred patients (76.6.1% versus 52.6%); however, the difference was not statistically significant (*P* =0.055).

***Conclusion:*** We found no significant different between operative mortality of transferred and non-transferred RAAA patients. Transfer of patients to tertiary centers with experienced vascular surgeons may delay the surgery. However, the transfer may be inevitable in areas where the optimal care of RAAA patients is not possible.

## Introduction

Ruptured abdominal aortic aneurysm (RAAA) is defined as the retroperitoneal or intraperitoneal leakage of the aneurysm sac. RAAA has had a mortality of up to 89% in western population-based studies.^1^ The natural history of RAAA is intraperitoneal or retroperitoneal bleeding, hypotension, hypovolemic shock, multi-organ failure and death.^2^ As a result, immediate vascular surgery consult and emergent surgery is recommended to prevent mortality.^3^ Many patients with RAAA are transferred to vascular surgery centers or to hospitals where experienced surgeons are available to manage RAAA. Thus, transferring RAAA patients may delay the surgery and increase the mortality.^4^ On the other hand, the lowest mortality for RAAA has been reported from teaching hospitals and academic centers.^5^

In the present study, we aimed to assess the in-hospital outcomes of patients with infrarenal RAAA in a high-volume and teaching vascular surgery center in Iran.

## Materials and Methods

### Patient Population

This study is a single-center retrospective analysis of patients with infrarenal RAAA during February 20, 2012 to December 21, 2019 at Shohada-Tajrish Medical Center, Shahid Beheshti University of Medical Sciences, Tehran, Iran. The inclusion criteria comprised patients with infrarenal RAAA and patients who arrived alive to the emergency department and to the operating table. The exclusion criteria comprised patients with pararenal and paravisceral AAA, patients who died at the emergency department and patients who did not arrive alive to the operating room.

### Background variables

The background variables included age, sex, past medical history (hypertension, coronary artery disease [CAD], hyperlipidemia, diabetes mellitus, congestive heart failure, and atrial fibrillation), smoking, and past medication history. In-hospital findings included the presenting signs (pain, abdominal mass, and loss of consciousness), hypotension at arrival, performance of radiological imaging, and operative findings. The operative findings included duration of operation, type of repair, use of bifurcated or tubular graft, first clamp site, and number of units of red packed cells and fresh frozen plasma during the operation.

### Management of Patients

All operations were performed at the same institution by either open surgery or EVAR. All open operations were performed by transperitoneal midline laparotomy incision and an infrarenal aortic cross clamp control was obtained when possible. When infrarenal control was not possible, subdiaphragmatic aortic compression device or supraceliac clamping was used. Alternatively, a Foley catheter from inside of the aneurysm sac was placed to obtain a more secure proximal control in a number of patients. Distal control was obtained on iliac arteries. Tubular or bifurcated Dacron grafts were used and sizing was performed intraoperatively based on the size of the proximal normal aorta. In emergent EVAR, prompt sizing was performed based on the preoperative computed tomography (CT) angiography.

### Endpoints

The patients were divided into two groups based on their transfer status (Transfer group versus non-transfer group). Transferred patients were transferred from another institution to the emergency department of Shohada-Tajrish Medical Center. The primary outcome of this study was in-hospital death. The secondary outcomes were in-hospital myocardial infraction (MI), abdominal compartment syndrome (ACS) and postoperative renal dysfunction requiring dialysis. The association of transfer status with primary and secondary outcomes were assessed. The association of background variables and in-hospital death was also assessed.

### Statistical analysis

Continuous variables were presented as mean ± standard deviation. Categorical variables were presented as frequency with a corresponding percentage. Univariate comparisons were evaluated with chi-square or Fisher’s exact test for categorical variables and independent sample student’s t-test for continuous variables, as needed. The statistical analysis was conducted by SPSS version 22.

## Results

We identified 66 patients with RAAA with consideration of the study period and inclusion/exclusion criteria. The mean age of the patients was 74.2 ± 8.3 years (Minimum = 54 and Maximum = 94). Eleven patients (16.7%) were female and 55 patients (83.3%) were male. Twelve patients (19.4%) had a history of an intact AAA. Forty-seven patients (71.2%) were transferred to the Shohada-Tajrish Medical Center from other institutions. The mean size of aneurysms was 85.9 ± 21.1 mm. Thirty patients (50.8%) underwent abdominal ultrasound, seven patients (12.1%) underwent abdominal CT, and 43 patients (71.7%) underwent abdominal CT angiography either at the index hospital or our institution. Two patients (3.0%) had history of previous EVAR. Table 1 illustrates the background variables and in-hospital findings in respect to the transfer status.

**Table 1:** Baseline characteristics and in-hospital findings of patients presenting with ruptured abdominal aortic aneurysms as modified by interfacility transfer

**Variable**	**Total** **N=66**	**Transferred** **N=47 (71.2%)**	**Non-Transferred** **N=19 (28.8%)**	***P*** ** Value**
Age (Years)	74.2 ± 8.3	74.2 ± 7.8	74.2 ± 7.5	0.996
Age ≥ 70 years	48 (72.7%)	34 (72.3%)	14 (73.7%)	0.912
Gender (Male)	55 (83.3%)	40 (85.1%)	15 (78.9%)	0.543
Hypertension	41 (66.1%)	31 (72.1%)	10 (52.6%)	0.136
Hyperlipidemia	18 (29.5%)	9 (21.4%)	9 (47.4%)	0.067
CAD	29 (46.8%)	19 (44.2%)	10 (52.6%)	0.539
DM	8 (12.9%)	6 (14.0%)	2 (10.5%)	0.711
CHF	6 (9.7%)	6 (14.0%)	0 (0.0%)	0.165
AF	2 (3.2%)	0 (0.0%)	2 (10.5%)	0.090
Smoking	28 (58.3%)	19 (57.6%)	9 (60.0%)	0.875
RAAS Inhibitor	20 (35.1%)	17 (42.5%)	3 (17.6%)	0.072
Diuretics	9 (15.8%)	7 (17.5%)	2 (11.8%)	0.587
Beta-Blockers	20 (35.1%)	14 (35.0%)	6 (35.3%)	0.983
Aspirin	20 (35.7%)	15 (35.8%)	5 (29.4%)	0.516
Clopidogrel	4 (7.0%)	3 (7.5%)	1 (5.9%)	0.827
Anticoagulants	3 (5.3%)	1 (2.5%)	2 (11.8%)	0.152
Pain	60 (93.8%)	43 (95.6%)	17 (89.5%)	0.358
Mass	8 (12.7%)	6 (13.6%)	2 (10.5%)	0.734
Loss of Consciousness	18 (28.6%)	14 (31.8%)	4 (21.1%)	0.385
SBP < 90 mm Hg	29 (43.9%)	19 (40.4%)	10 (52.6%)	0.366
Abdominal US	30 (50.8%)	21 (52.5%)	9 (47.4%)	0.713
CT Angiography	43 (71.7%)	29 (70.7%)	14 (73.7%)	0.813
Repair Type Open EVAR	63 (95.5%) 3 (6.4%)	44 (93.6%) 3 (4.5%)	19 (100.0%) 0 (0.0%)	0.260
Operative Time	185.2 ± 62.8	185.0 ± 62.0	185.2 ± 63.8	0.990
Graft type^a^ Tubular Bifurcated	32 (55.2%) 26 (44.8%)	21 (51.2%) 20 (48.8%)	11 (64.7%) 6 (35.3%)	0.347
First Clamp site^a^ Infrarenal Supraceliac Compression Thoracotomy	36 (61.0%) 15 (25.4%) 7 (11.9%) 1 (1.7%)	22 (55.0%) 13 (32.5%) 5 (12.5%) 0 (0.0%)	14 (73.7%) 2 (10.5%) 2 (10.5%) 1 (5.3%)	0.149
Intra-aneurysmal control^b^	5 (8.5%)	4 (10.0%)	1 (5.3%)	0.542
PC units^c^	4.9 ± 2.7	4.9 ± 2.5	4.9 ± 3.1	0.923
FFP units^c^	3.0 ± 2.3	3.0 ± 2.2	2.9 ± 2.7	0.873

Abbreviationas: AF, atrial fibrillation; CAD, coronary artery disease; CHF, congestive heart failure; CT, computed tomography; DM, diabetes mellitus, FFP, fresh frozen plasma; PC, packed cell; RAAS, renin angiotensin aldosterone system; SBP, systolic blood pressure; US, ultrasound; EVAR, endovascular aneurysm repair ^a^The frequencies are for open repairs. ^b^By a Foley Catheter (24 F) ^c^Numbers represent the perioperative administration of PC and FFP. Postoperative administration of blood products in intensive care unit is not calculated in this table.

**Figure 1:**
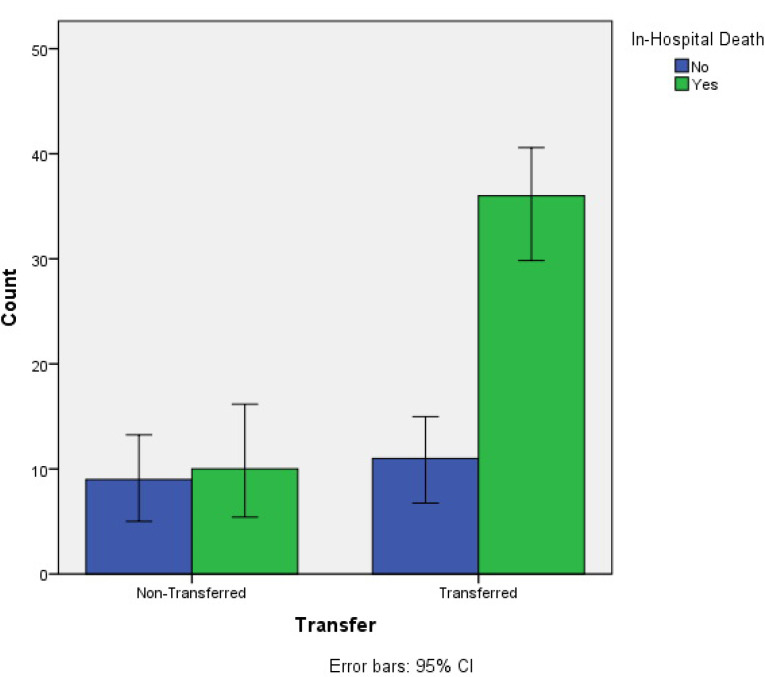
The association of in-hospital death and transfer (Univariate analysis; *P=0.055*). *CI*, Confidence Interval

There were 46 in-hospital deaths (69.7%) and three in-hospital MIs (4.5%). Three patients (4.5%) had postoperative ACS and six patients (9.1%) had postoperative renal dysfunction requiring dialysis. Transfer patients had an increased rate of in-hospital death compared to non-transferred patients (76.6.1% versus 52.6%); however, the difference was not statistically significant (*P=0.055*; Figure 1). Table 2 tabulates the in-hospital events in respect to the transfer status.

**Table 2:** In-hospital outcomes in patients presenting with ruptured abdominal aortic aneurysms (Transferred versus Non-Transferred)

**Outcome**	**Total** **N=66**	**Transferred** **N=47 (71.2%)**	**Non-Transferred** **N=19 (28.8%)**	***P*** ** Value**
In-Hospital Death Death before Induction Intraoperative death Postoperative death	46 (69.7%) 2 (3.3%) 10 (15.2%) 34 (51.5%)	36 (76.6%) 2 (4.3%) 8 (17.0%) 26 (55.3%)	10 (52.6%) 0 (0.0%) 2 (10.5%) 8 (42.1%)	0.055
In-Hospital MI	3 (4.5%)	2 (4.3%)	1 (5.3%)	0.646
ACS	3 (4.5%)	3 (6.4%)	0 (0.0%)	0.354
Renal dysfunction^a^	6 (9.1%)	6 (12.8%)	0 (0.0%)	0.118

Abbreviation: ACS, abdominal compartment syndrome; MI, myocardial infarction ^a^Necessitating postoperative dialysis

Patients who died were compared with patients who survived in respect to the background variables and in-hospital findings. Death was associated with a number of variables in univariate analysis. These included age ≥ 70 years old, systolic blood pressure < 90 mm Hg on arrival to the emergency department, unconsciousness on arrival to the emergency department, use of supra-renal clamp and infusion of more red packed cells. Table 3 demonstrates the background variables and in-hospital findings as stratified by in-hospital death.

**Table 3:** Baseline characteristics and in-hospital findings of patients presenting with ruptured abdominal aortic aneurysmsas modified by in-hospital death

**Variable**	**In-Hospital Death**		***P*** ** Value**
	**Yes (N=46)**	**No (N=20)**	
Age ≥ 70 years	37 (80.4%)	11 (55.5%)	*0.033*
Gender (Male)	38 (82.6%)	17 (85.0%)	0.811
Hypertension	31 (73.8%)	10 (50.0%)	0.064
Hyperlipidemia	11 (26.2%)	7 (36.8%)	0.398
CAD	21 (50.0%)	8 (40.0%)	0.461
DM	6 (14.3%)	2 (10.0%)	0.638
CHF	3 (7.1%)	3 (15.0%)	0.328
AF	1 (2.4%)	1 (5.0%)	0.585
Smoking	16 (50.0%)	12 (75.0%)	0.098
RAAS Inhibitor	14 (37.8%)	6 (30.0%)	0.554
Diuretics	4 (10.8%)	5 (25.0%)	0.161
Beta-Blockers	14 (37.8%)	6 (30.0%)	0.554
Aspirin	13 (35.1%)	7 (36.8%)	0.900
Clopidogrel	1 (2.7%)	3 (15.0%)	0.083
Anticoagulants	1 (2.7%)	2 (10%)	0.239
Pain	40 (90.9%)	20 (100.0%)	0.164
Mass	5 (11.4%)	3 (15.8%)	0.628
Loss of Consciousness	16 (36.4%)	2 (10.5%)	*0.037*
SBP < 90 mmHg	24 (52.2%)	5 (25.0%)	*0.041*
Abdominal US	24 (60.0%)	6 (31.6%)	*0.041*
CT Angiography	29 (70.7%)	14 (73.7%)	0.813
Repair Type Open EVAR	44 (95.7%) 2 (4.3%)	19 (95.0%) 1 (5.0%)	0.907
Operative Time	176.4 ± 58.8	204.0 ± 68.4	0.129
Graft type^a^ Tubular Bifurcated	20 (51.3%) 19 (48.7%)	12 (63.2%) 7 (36.8%)	0.393
First Clamp site^a^ Infrarenal Supraceliac Compression Thoracotomy	21 (50.0%) 13 (31.0%) 7 (16.7%) 1 (2.4%)	15 (88.2%) 2 (11.8%) 0 (0.0%) 0 (0.0%)	*0.048*
Intra-aneurysmal control^b^	5 (11.9%)	0 (0.0%)	0.137
PC units^c^	5.4 ± 2.6	3.9 ± 2.6	*0.035*
FFP units^c^	3.1 ± 2.3	2.7 ± 2.3	0.463

AF, atrial fibrillation; CAD, coronary artery disease; CHF, congestive heart failure; CT, computed tomography; DM, diabetes mellitus, FFP, fresh frozen plasma; PC, packed cell; RAAS, renin angiotensin aldosterone system; SBP, systolic blood pressure; us, ultrasound; EVAR, endovascular aneurysm repair ^a^The frequencies are for open repairs. ^b^By a Foley Catheter (24 F) ^c^Numbers represent the perioperative administration of PC and FFP. Postoperative administration of blood products in intensive care unit is not calculated in this table.

## Discussion

We found that transfer of RAAA patients is associated with an increased in-hospital mortality rate (76.6.1% versus 52.6%) but the difference was not statistically significant (*P* > 0.05). In-hospital MI, ACS and postoperative dialysis were not associated with transfer of the patients as well (*P* > 0.05). We identified 66 patients within the study period and multivariate analysis was not applicable due to the small patient population.

Karthikesalingam et al studied the mortality of RAAA in England and US during 2005 to 2010. They found that increased use of endovascular repair, high hospital volume and bed, teaching hospitals, and admission on a weekday are associated with reduced mortality.^5^ Qiu et al reviewed 56 consecutive patients with RAAA in China. They revealed that in-hospital mortality is increased in patients who transferred from another institution (68.8% versus 33.3%; *P* < 0.001).^4^ We found no significant difference in mortality of transferred and non-transferred RAAA patients. Management of RAAA necessities vascular surgery team and intensive unit care beds that may not be available in all hospitals. Thus, transfer of most RAAA patients is inevitable despite the probable increased risk of ongoing leak and death.

In contrast to the probable effect of transfer on outcomes of RAAA, a number of studies demonstrate lower mortality rates in both elective and ruptured AAAs when performed in high volume and teaching centers.^5,6^ This peri-operative advantage in high volume centers is more pronounced in open RAAA surgery and individual surgeon case load does not have an impact on outcomes.^7^ Additionally, the introduction of EVAR has changed the management of RAAA dramatically and EVAR is considered the first-line treatment of RAAA in many institutions.^8-11^ Thus, unavailability of EVAR at low-volume and non-tertiary hospitals may further centralize RAAA patients to teaching and tertiary level institutions and the influence of transfer should be considered in these circumstances as well.

Mell et al studied 4439 patients with RAAA during 2005 to 2010 in three states of the US. There were 19.1% transferred patients in their series. They found that older age, private insurance, and comorbidities were associated negatively with transfer in multivariate analysis. They revealed that transfer was associated with a lower operative mortality (but an increased overall mortality when including transferred patients who died without surgery).^12^ We did not find a significant increase in the operative mortality of transferred patients; however, we did not include the patients who died at the emergency department.

The weekend and holidays may also have an impact on outcomes of RAAA. According to study of O’Donnell et al transfer of RAAA patients in weekends leads to a higher mortality than the transfers during the weekdays. Additionally, they found that transfer of RAAA patients are more common in weekends.^13^ We did not find a significant association between operative mortality and transfer. However, we do not have any data on outcome of RAAA patients in the index hospitals in Iran. We believe that transfer of RAAA patients may delay the surgical treatment; however, the unavailability of the vascular surgery service at index hospitals makes transfers inevitable in most circumstances.

Despite the fact that the unavailability of vascular surgery team and intensive care facilities make transfer of RAAA patients inevitable, some investigators report encouraging results of RAAA management at district general hospitals.^14^ It seems that the best place for the management of RAAA is the index hospital provided that the surgical team are proficient enough to manage the patients. This strategy may shorten the time from diagnosis to surgery and improve the outcomes.

The application of preoperative imaging modalities in RAAA is controversial. Many authors believe that application of preoperative CT angiography does not delay surgery in hemodynamically stable patients.^15^ Moreover, it is essential for emergent performance of EVAR.^16^ However, it may be challenging in patients with severe hypotensive shock and in patients with acute renal failure resulting from profound hypovolemia. Many patients undergo ultrasound on their initial evaluations. According to Reed et al the application of ultrasound accelerates the diagnosis of RAAA in the emergency department.^17^ We found that 50.8% of our patients underwent preoperative abdominal ultrasound. Interestingly, we found a significant association between operative mortality and performance of abdominal ultrasound. This may result from delay of surgery due to the time required for conducting sonography.

Zdanowski et al studied the outcomes of RAAA at the university and county hospitals. Their patients were operated on by the same vascular surgeon. They found that on-table mortality for patients with ruptured AAA and shock was 12% at the university hospitals and 15% at the county hospitals. They believe that the mortality is significantly higher if the operation is delayed by more than 45 minutes.^18^ Thus, the journey of vascular surgeon to a county hospital to operate a patient with RAAA should be considered only when it is assured that this strategy does not cause more delays than the transfer of the patient. In addition, this journey should be done by a vascular surgeon other than the vascular surgeon who is on-call at the teaching hospital.

Hames et al conducted a single-institutional study in Canada to assess the effect of patient transfers on outcomes of RAAA. They found that 70.4% of their RAAA patients was transferred from other institutions. They revealed that transferred patients took twice as long as direct patients to get to the operating room; however, the transfer of patients did not increase the mortality significantly.^19^ In another single institutional study from the US, Vogel et al found an overall mortality rate of 67% for patients with RAAA. They reported mortality rates of 69% and 65%, for non-transferred and transferred patients, respectively.^20^ We found in-hospital mortality rates of 76.6.1% and 52.6% for our transferred and non-transferred patients, respectively.

The overall mortality rate of RAAAs was high in the present study (69.7%). This is somehow due to the catastrophic nature of RAAA which leads to severe hypovolemic shock, multi-organ failure and death. The main issue in this respect is the delays that occur before the transfer and after the admission of patients to a vascular surgery center. Thus, both transfer delays and door to intervention delays play important roles in increasing the mortality. However, we did not have accurate data on the transfer and door to intervention times. Additionally, this study is limited in terms of the small sample size and reliance on univariate analysis. The potential for coding errors and missing data persists and these are inherent to any study using patients’ records.

## Conclusion

In conclusion, RAAA is a catastrophic condition with high mortality. RAAA necessitates prompt vascular surgery consult and surgical intervention. Transfer of patients to tertiary centers with experienced vascular surgeons may delay the surgery. However, the transfer may me inevitable in areas where the optimal care of RAAA patients is not available.

## Acknowledgements

We appreciate Dr. Behnam Honarvar from the Health Policy Research Center, Shiraz University of Medical Sciences, Shiraz, Iran for his invaluable comments and support during this study. We also thank Dr. A. Shahnaee for her kind helps during the preparation of this article.

## Funding

None.

## Ethical approval

Shahid Beheshti University of Medical Sciences: IR.SBMU.RETECH.REC.1400.934; Shiraz University of Medical Sciences: IR.SUMS.REC.1399.1226

## Competing interests

The authors have no conflicts of interests.

## References

[1] Lindsay TF. Ruptured aortoiliac aneurysms and their management. In: Sidawy AN, Perler BA, eds. Rutherford’s Vascular Surgery and Endovascular Therapy. 9th ed. Philadelphia, PA: Elsevier; 2019. p. 944-946.

[2] Maziak DE, Lindsay TF, Marshall JC, Walker PM (1998). The impact of multiple organ dysfunction on mortality following ruptured abdominal aortic aneurysm repair. Ann Vasc Surg.

[3] Starnes BW, Quiroga E, Hutter C, Tran NT, Hatsukami T, Meissner M (2010). Management of ruptured abdominal aortic aneurysm in the endovascular era. J Vasc Surg.

[4] Qiu J, Zhou W, Zhou W, Tang X, Yuan Q, Xiong J (2016). The beneficial place for the treatment of ruptured abdominal aortic aneurysms. Int J Surg.

[5] Karthikesalingam A, Holt PJ, Vidal-Diez A, Ozdemir BA, Poloniecki JD, Hinchliffe RJ (2014). Mortality from ruptured abdominal aortic aneurysms: clinical lessons from a comparison of outcomes in England and the USA. Lancet.

[6] Holt PJ, Poloniecki JD, Gerrard D, Loftus IM, Thompson MM (2007). Meta-analysis and systematic review of the relationship between volume and outcome in abdominal aortic aneurysm surgery. Br J Surg.

[7] Kontopodis N, Galanakis N, Akoumianakis E, Ioannou CV, Tsetis D, Antoniou GA (2021). Editor’s choice-systematic review and meta-analysis of the impact of institutional and surgeon procedure volume on outcomes after ruptured abdominal aortic aneurysm repair. Eur J Vasc Endovasc Surg.

[8] Steenberge SP, Sorour AA, Sundaram A, Bena J, Kirksey L (2021). Comparative outcomes of open abdominal therapy after ruptured abdominal aortic aneurysm via open and endovascular approaches. Ann Vasc Surg.

[9] D’Oria M, Gunnarsson K, Wanhainen A, Mani K. Long-term survival after repair of ruptured abdominal aortic aneurysms is improving over time - nationwide analysis during twenty-four years in Sweden (1994-2017). Ann Surg. 2021. 10.1097/sla.0000000000005030 34183511

[10] Alsusa H, Shahid A, Antoniou GA. A comparison of endovascular versus open repair for ruptured abdominal aortic aneurysm - meta-analysis of propensity score-matched data. Vascular. 2021:17085381211025168. 10.1177/17085381211025168 34126813

[11] Briggs CS, Sibille JA, Yammine H, Ballast JK, Anderson W, Nussbaum T (2018). Short-term and midterm survival of ruptured abdominal aortic aneurysms in the contemporary endovascular era. J Vasc Surg.

[12] Mell MW, Wang NE, Morrison DE, Hernandez-Boussard T (2014). Interfacility transfer and mortality for patients with ruptured abdominal aortic aneurysm. J Vasc Surg.

[13] O’Donnell TFX, Li C, Swerdlow NJ, Liang P, Pothof AB, Patel VI (2019). The weekend effect in AAA repair. Ann Surg.

[14] Lee CH, Chang CJ, Huang JK, Yang TF (2016). Clinical outcomes of infrarenal abdominal aortic aneurysms that underwent endovascular repair in a district general hospital. J Thorac Dis.

[15] Lloyd GM, Bown MJ, Norwood MG, Deb R, Fishwick G, Bell PR (2004). Feasibility of preoperative computer tomography in patients with ruptured abdominal aortic aneurysm: a time-to-death study in patients without operation. J Vasc Surg.

[16] Mehta M (2010). Endovascular aneurysm repair for ruptured abdominal aortic aneurysm: the Albany Vascular Group approach. J Vasc Surg.

[17] Reed MJ, Cheung LT (2014). Emergency department led emergency ultrasound may improve the time to diagnosis in patients presenting with a ruptured abdominal aortic aneurysm. Eur J Emerg Med.

[18] Zdanowski Z, Danielsson G, Jonung T, Kaij J, Ribbe E, Sahlin C (2002). Outcome of treatment of ruptured abdominal aortic aneurysms depending on the type of hospital. Eur J Surg.

[19] Hames H, Forbes TL, Harris JR, Lawlor DK, DeRose G, Harris KA (2007). The effect of patient transfer on outcomes after rupture of an abdominal aortic aneurysm. Can J Surg.

[20] Vogel TR, Nackman GB, Brevetti LS, Crowley JG, Bueno MM, Banavage A (2005). Resource utilization and outcomes: effect of transfer on patients with ruptured abdominal aortic aneurysms. Ann Vasc Surg.

